# Tissue-specific 5-hydroxymethylcytosine landscape of the human genome

**DOI:** 10.1038/s41467-021-24425-w

**Published:** 2021-07-12

**Authors:** Bo He, Chao Zhang, Xiaoxue Zhang, Yu Fan, Hu Zeng, Jun’e Liu, Haowei Meng, Dongsheng Bai, Jinying Peng, Qian Zhang, Wei Tao, Chengqi Yi

**Affiliations:** 1 State Key Laboratory of Protein and Plant Gene Research, School of Life Sciences, Peking University, Beijing, China; 2 Peking-Tsinghua Center for Life Sciences, Academy for Advanced Interdisciplinary Studies, Peking University, Beijing, China; 3 Key Laboratory of Cell Proliferation and Differentiation, School of Life Sciences, Peking University, Beijing, China; 4 Peking University-Tsinghua University-National Institute of Biological Sciences Joint Graduate Program (PTN), Peking University, Beijing, China; 5 Department of Urology, Peking University First Hospital, Beijing, China; 6 Institute of Urology, Peking University, Beijing, China; 7 National Urological Cancer Center, Beijing, China; 8 Peking University Binhai Hospital, Tianjin, China; 9 Department of Chemical Biology and Synthetic and Functional Biomolecules Center, College of Chemistry and Molecular Engineering, Peking University, Beijing, China

**Keywords:** DNA, DNA methylation, Epigenomics

## Abstract

5-Hydroxymethylcytosine (5hmC) is an important epigenetic mark that regulates gene expression. Charting the landscape of 5hmC in human tissues is fundamental to understanding its regulatory functions. Here, we systematically profiled the whole-genome 5hmC landscape at single-base resolution for 19 types of human tissues. We found that 5hmC preferentially decorates gene bodies and outperforms gene body 5mC in reflecting gene expression. Approximately one-third of 5hmC peaks are tissue-specific differentially-hydroxymethylated regions (tsDhMRs), which are deposited in regions that potentially regulate the expression of nearby tissue-specific functional genes. In addition, tsDhMRs are enriched with tissue-specific transcription factors and may rewire tissue-specific gene expression networks. Moreover, tsDhMRs are associated with single-nucleotide polymorphisms identified by genome-wide association studies and are linked to tissue-specific phenotypes and diseases. Collectively, our results show the tissue-specific 5hmC landscape of the human genome and demonstrate that 5hmC serves as a fundamental regulatory element affecting tissue-specific gene expression programs and functions.

## Abstract (web summary)

Charting the landscape of 5hmC in human tissues is fundamental to understanding its regulatory functions. Here, we systematically profiled the whole-genome 5hmC landscape at single-base resolution for 19 types of human tissues and found 5hmC shows tissue-specific patterns.

## Introduction

DNA methylation at the fifth position of cytosine (5mC), which is established and maintained by DNA methyltransferases, is a predominant epigenetic modification that is critical for various biological and pathological processes, including silencing of transposable elements, regulation of gene expression, genomic imprinting and X-chromosome inactivation^1,2^. 5-Hydroxymethylcytosine (5hmC), also known as the “sixth base” of DNA, was discovered as another relatively abundant form of cytosine modification in Purkinje neurons and mouse embryonic stem cells (mESCs)^3,4^. Further studies found that the ten-eleven translocation (TET) family of Fe(II)- and α-ketoglutarate-dependent DNA dioxygenases (including TET1, TET2, and TET3) catalyzes the sequential oxidation of 5mC to 5hmC, 5-formylcytosine (5fC) and 5-carboxylcytosine (5caC)^4–6^. Subsequently, the DNA repair enzyme thymine-DNA glycosylase (TDG) can excise 5fC and 5caC to generate abasic sites, which eventually results in the regeneration of unmodified cytosines by the base excision repair pathway^6–8^. This TET-TDG pathway is known as the active DNA demethylation pathway.

In addition to being an intermediate of 5mC oxidation, evidence shows that 5hmC is a stable epigenetic mark with regulatory functions^9^. The specific genomic distribution pattern of 5hmC, such as its high enrichment in the gene bodies of transcriptionally active genes, promoters and enhancers, hints at specific biological roles of 5hmC^10–12^. In addition, the 5hmC level undergoes highly dynamic changes during development, differentiation and cancer^4,13,14^. For instance, the global 5hmC content is dramatically reduced in multiple human cancers compared with that in the normal tissues adjacent to the cancer^15–17^, suggesting that dysregulation of genomic 5hmC may be involved in tumorigenesis. Mechanistically, abnormal hydroxymethylation status impacts chromatin structure by interrupting the interaction of 5hmC-specific binding proteins with 5hmC^18^. Altogether, existing data have suggested a critical role of 5hmC in developmental processes and an association of dysregulation of 5hmC with human diseases.

Different methods have been developed to map the genomic distribution of 5hmC, including affinity-based methods (5hmC-DIP-Seq, CMS-Seq, GLIB, and hMe-Seal) and high-resolution methods (oxBS-Seq, TAB-Seq, hmC-CATCH, TAPS, and ACE-Seq)^19,20^. For instance, we previously developed hmC-CATCH^21^, which is a bisulfite-free method for genome-wide detection of 5hmC. hmC-CATCH couples selective chemical labeling and biotin pulldown^21–24^, thus allowing 5hmC enrichment and detection at single-base-resolution. While hydroxymethylome maps have been obtained for mammalian cell lines and mouse tissues, 5hmC profiles in human tissues have not been as comprehensively characterized.

In this study, we generated genome-wide, base-resolution 5hmC data via hmC-CATCH across 19 human tissue types, represented by 60 tissue samples derived from 6 Chinese donors. We found that 5hmC is enriched in gene bodies and that it exhibits a better positive correlation with gene expression than gene body 5mC. Tissue-specific differentially hydroxymethylated regions (tsDhMRs) reside in regions enrich for regulatory elements, and co-localize with transcription factor-binding sites, which may regulate the tissue-specific gene expression network. Furthermore, tsDhMRs were shown to enrich disease-related single-nucleotide polymorphisms (SNPs) identified by genome-wide association studies (GWAS), linking 5hmC to human phenotypes and pathologies. Collectively, our results provide a high-resolution, high-quality atlas of DNA hydroxymethylation across diverse human tissues and provide a resource for exploring the role of this modification in gene expression networks.

## Results

### 5-Hydroxymethylcytosine landscape of diverse human tissues

**Fig. 1:**
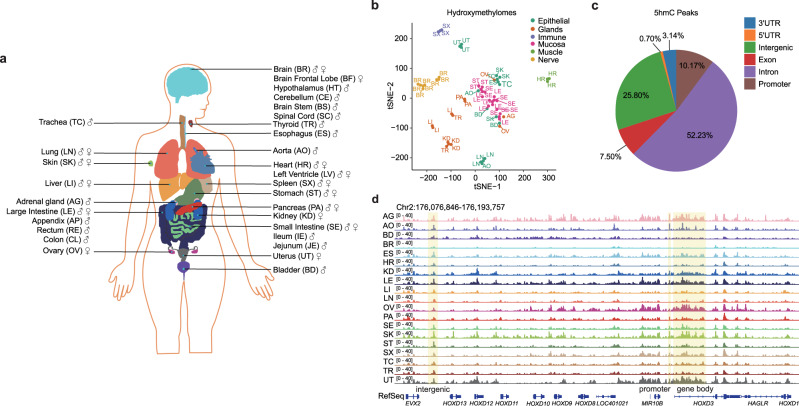
Hydroxymethylation landscape of human tissues. **a** Human tissues analyzed in this study. Samples are denoted by the two-letter code in parentheses. Colors indicate each tissue type. **b** tSNE cluster of all tissues using the global 5hmC signals in 10-kb bins. **c** Pie chart showing the percentage of 5hmC peaks in each class of genomic features. The promoter regions are defined as 2 kb around the TSS. **d** IGV visualization of the 5hmC signals surrounding the HOXD gene cluster on chromosome 2. 5hmC signals in promoter, gene body or intergenic region are highlighted.

To systematically investigate the dynamics of DNA hydroxymethylation across different human tissues, we collected samples of 19 tissue types from 6 Chinese donors: 3 males and 3 females (Fig. 1a and Supplementary Tables 1 and 2). We profiled the 5hmC signals of the various tissues via hmC-CATCH (60 hmC-CATCH samples; 83–183 million paired-end reads per sample; average, 128 million paired-end reads) (Supplementary Table 3). We found that the 5hmC-containing spike-in sequence was specifically and efficiently enriched (Supplementary Fig. 1a) and displayed a high detection rate for 5hmC (~91% and ~98% before and after pulldown) (Supplementary Fig. 1b, c and Supplementary Table 4), both of which showed the high quality of the hydroxymethylome generated by hmC-CATCH. We found that ~80% reads in the pulldown samples have C-to-T conversion and ~94% of peaks contain at least one confident 5hmC site; nevertheless, in order to ensure the confidence of detection, we only selected the reads that contain C-to-T conversion signals (Supplementary Fig. 1d, e). This made sure that all peaks are 5hmC-enriched regions. It is also worth mentioning that after removing reads without C-to-T conversion, ~97% peaks were still identified. In addition, tissues derived from the same organ system from different donors were clearly clustered together (Fig. 1b and Supplementary Fig. 1f), further demonstrating the confidence of the 5hmC data.

We next analyzed the genomic features of 5hmC. We identified 721,404 reproducible 5hmC peaks, most of which were located in intron and intergenic regions (Fig. 1c). The peak number of each tissue type ranged from 272,391 to 525,080 (Supplementary Fig. 1g). We found that 5hmC was highly enriched in gene bodies, especially in exon regions, while it was depleted in intergenic regions (Supplementary Fig. 1h). Taking the HOXD gene cluster as an example, we observed 5hmC signals in distal regulatory elements, promoters and gene bodies; such signals were also dynamic among tissues (Fig. 1d). Hence, via hmC-CATCH, we were able to generate the whole-genome 5hmC landscape of different human tissues.

### Hydroxymethylome at single-base-resolution

**Fig. 2:**
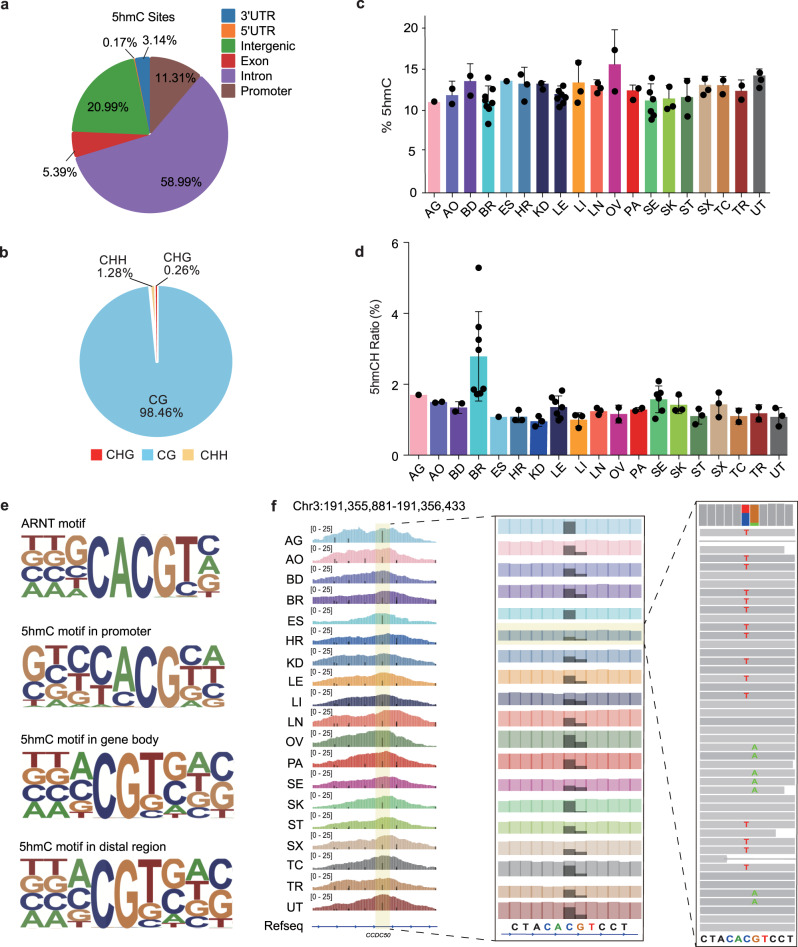
Single-base-resolution profiles of 5hmC. **a** Pie chart showing the percentage of 5hmC sites in each class of genomic features. **b** Distribution of 5hmCG, 5hmCHH and 5hmCHG sites. **c** Average proportion of symmetrically distributed 5hmC sites (Data are represented as mean values + /− SD). The number of samples are shown in Supplementary Table 2. **d** Proportions of 5hmCH in different tissues (Data are represented as mean values + /− SD). The number of samples are shown in Supplementary Table 2. **e** The most significant 5hmC motif in different genomic elements demonstrates partial sequence overlap with the known transcription factor motif of ARNT. **f** IGV visualization of the 5hmC site signals on chromosome 3. The gray bar represents the C-to-T counts. 5hmC is asymmetrical at the 5hmCG site.

To obtain a more detailed and clearer landscape of the hydroxymethylome, we analyzed 5hmC sites at single-base-resolution. We identified 9,416,937 reproducible 5hmC sites, with the site number of each tissue type ranging from 1,217,850 to 2,429,878 (~3–10% of CpG sites were hydroxymethylated) (Supplementary Fig. 2a). While the number of 5hmC peaks in the brain is comparable to other tissues (Supplementary Fig. 1g, presumably due to the insensitivity of enrichment-based methods to overall modification level change), the number of 5hmC sites in the brain is significantly higher than that in other tissues (*P*-value: 6.57 × 10^−7^), consistent with the higher 5hmC content in the brain^20^. A representative locus is shown in Supplementary Fig. 2b, in which the single 5hmC sites identified by hmC-CATCH co-localize nicely with 5hmC sites by TAB-seq. More than half of the 5hmC sites were identified in at least two tissues, suggesting that a substantial number of 5hmC sites could be conserved (Supplementary Fig. 2c). Most of the 5hmC sites were located in intron and intergenic regions (Fig. 2a) and were highly enriched in gene bodies (Supplementary Fig. 2d).

5mC in the CG context is almost symmetrical due to the maintenance of DNA methyltransferase 1. A total of 98.46% of the 5hmC sites identified were located in the CG context (Fig. 2b), but only 13% of the 5hmCG sites were symmetrical (Fig. 2c), which is consistent with previous data in mESCs^25^. Thus, this pattern of 5hmC differs from that of 5mC, which is symmetrical in human tissues^26^. In addition, the proportion of 5hmC in the non-CG context (5hmCH) was variable in different tissues, ranging from 0.96% to 2.79% (Fig. 2d and Supplementary Fig. 2e). Previous research suggests that DNA methylation is rapidly accumulated in 5mCH sites during synaptogenesis^27^, and we also found a higher 5hmCH ratio in the brain than that in other tissues (Fig. 2d and Supplementary Fig. 2e), indicating that 5hmCH may play a role during brain development.

We next analyzed the 5hmC context in tissues and found a “CA^hm^CGT” motif for 5hmC (Fig. 2e and Supplementary Fig. 2f, g), which coincided with the binding site of ARNT, a housekeeping gene that participates in important metabolic processes. Within ARNT ChIP-seq signals, cytosines are hydroxymethylated in most tissues, with evident C-to-T conversion in the sequencing reads and hence clear single 5hmC sites (Fig. 2f and Supplementary Fig. 2h). This observation hints that 5hmC may positively influence the genomic occupancy of ARNT. Notably, it has been reported that the ARNT binding motif loses 5hmC signals in esophageal cancer patients compared to healthy individuals^28^. With regard to the 5hmCH modification, we found that the most frequent base following 5hmCH was adenine (Supplementary Fig. 2i, j), consistent with the 5mCH sequence preference^26^.

Taken together, the results of the base-resolution hydroxymethylome analysis presented above revealed the varied 5hmCH ratio among tissues, the asymmetrical distribution of 5hmCG and the sequence preference of 5hmC, providing a potential mechanism for how 5hmC can be modified, recognized and dynamically regulated.

### Gene body 5hmC excels 5mC in reflecting gene expression

**Fig. 3:**
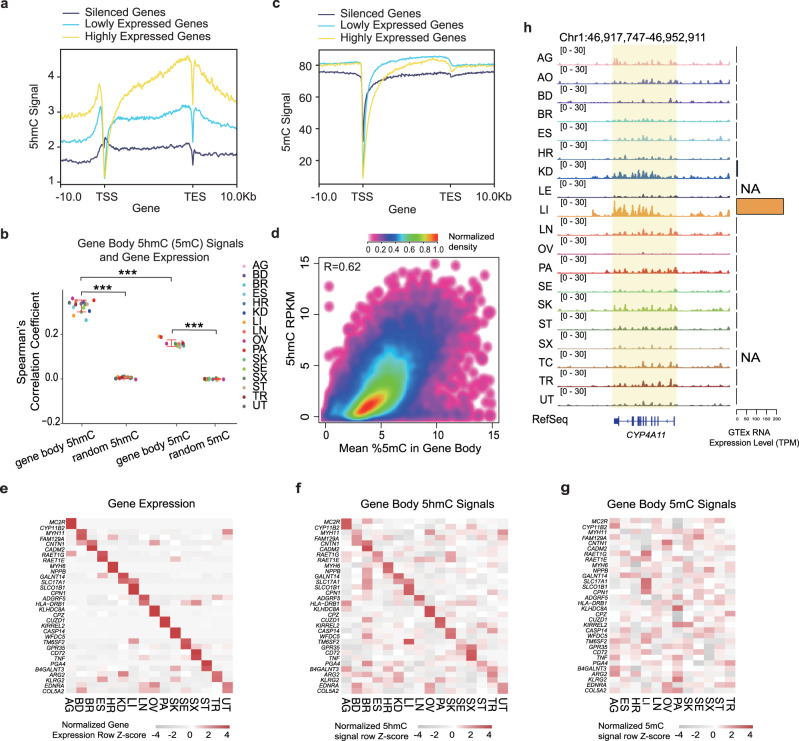
Gene body 5hmC correlates well with gene expression in human tissues. **a** 5hmC profiles of genes expressed at high (yellow), low (cyan), and silenced (blue) levels in heart tissue. **b** Spearman’s correlation of gene body 5hmC (5mC) signals and gene expression levels. Random regions were selected as controls (***represents *P*-value < 0.0001; two-side Mann–Whitney *U*-test; *n*_5hmC_ = 16 tissue types; *n*_5mC_ = 12 tissue types; 5hmC vs. random *P*-value: 3.32 × 10^−9^;5mC vs. random *P*-value: 7.396 × 10^−7^ 5hmC vs. 5mC *P*-value :6.57 × 10^−8^; red lines represent the first and third quantiles). **c** 5mC profiles of genes expressed at high (yellow), low (cyan) and silenced (blue) levels in heart tissue. **d** Scatter plot showing the correlation of 5mC and 5hmC signals at gene bodies. **e** Heatmap displaying the expression levels of tissue-specific marker genes. Gene expression of matched tissue samples downloaded from the GTEx project. **f**, **g** Heatmap displaying normalized gene body 5hmC (**f**) and 5mC (**g**) signals of tissue-specific marker genes (order of rows as in **e**). 5mC data downloaded from ENCODE. **h** IGV visualization of the 5hmC signals at the gene body of *CYP4A11* and surrounding regions. Gene expression levels of the gene in different tissues are shown on the right.

As the 5hmC level in the gene body shows a positive correlation with gene expression in mESCs and the mouse brain^18^, we analyzed whether the gene body 5hmC level in human tissues also corresponds to gene expression. Indeed, we found that stronger gene body 5hmC signals were correlated with higher gene expression levels (Fig. 3a and Supplementary Fig. 3a). For instance, the genes that escape X chromosome inactivation display higher gene body 5hmC levels than the inactive genes (Supplementary Fig. 3b, c). As the gene body 5mC level is reported to be positively correlated with transcription^1^, we also compared the gene body 5mC and 5hmC levels (Fig. 3b, c). Although 5mC co-localized with 5hmC in gene bodies (Fig. 3d), we found that the gene body 5hmC level exhibited a stronger quantitative correlation with gene expression levels than the gene body 5mC level. More specifically, we found 5hmC is almost linearly positively correlated with gene expression, while 5mC shows a non-monotonic and bell-shaped relationship with gene expression (Supplementary Fig. 4a–e)^29^.

To further explore the dynamics of the gene body 5hmC across human tissues, we defined 4,031 tissue-specifically expressed genes using RNA expression data generated by the Genotype-Tissue Expression project (GTEx)^30^ (Supplementary Fig. 4f). The expression of tissue-specific genes and gene body 5hmC levels also demonstrated a positive correlation, which was absent for 5mC (Supplementary Fig. 4g–i). Correlations of representative tissue-specific marker genes are shown in Fig. 3e–g. For instance, *PGA4* is specifically expressed in the stomach and encodes the precursor of pepsin; we found the highest level of gene body 5hmC signals in the stomach among all tissues (Fig. 3e, f). In contrast, gene body 5mC failed to display a positive correlation with tissue-specific gene expression (Fig. 3g). Taking *CYP4A11* as another example showed the following: this gene is specifically expressed in the liver and is involved in drug metabolism and the synthesis of cholesterol; consistent with this function, we also found higher gene body 5hmC levels in the liver than in other tissues (Fig. 3h). Again, this pattern was not found for 5mC (Supplementary Fig. 4j). As 5hmC shows a strong co-localization with Poll^21^, it was proposed to play a role in the transcription process. Collectively, these data show that the gene body 5hmC level correlates well with gene expression and may be associated with the maintenance of tissue-specific functions.

### tsDhMRs reside in regions that may function as tissue-specific regulatory elements

**Fig. 4:**
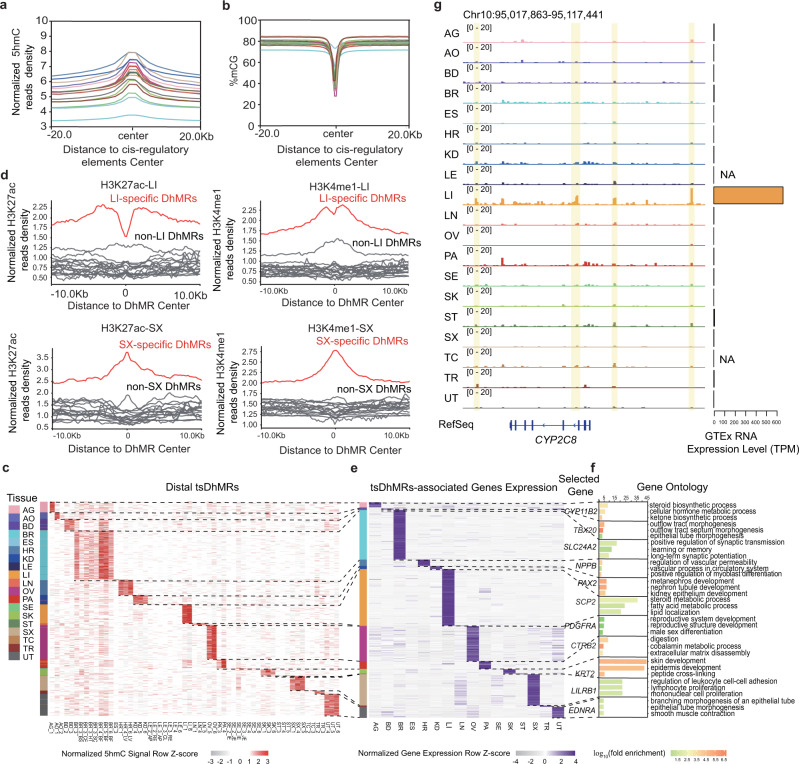
tsDhMRs reside in regions with tissue-specific regulatory elements. **a**, **b** Profiles of 5hmC (**a**) and 5mC (**b**) signals in their corresponding distal regulatory elements. The distal regulatory elements defined by the ENCODE Project Consortium. **c** Heatmap showing the normalized 5hmC signals in distal tsDhMRs. **d** Profiles of H3K27ac and H3K4me1 modifications around distal tsDhMRs in the liver (LI) and spleen (SX). Red represents the tissue type mentioned in the title of each panel and gray denotes the rest of the tissue types. **e** Heatmap showing the expression of genes associated with distal tsDhMRs within 500 kb. **f** GO enrichment and representative genes of distal tsDhMR-associated genes. **g** IGV visualization of the 5hmC signals near *CYP2C8* on chromosome 10. The highlighted regions are liver-specific DhMRs.

5hmC may participate in development and cell differentiation^11,31,32^; nevertheless, its regulatory functions in different tissues have not been reported. We found 5hmC showed high enrichment in cis-regulatory elements in the corresponding tissues (Fig. 4a). In contrast, 5mC are depleted in cis-regulatory elements (Fig. 4b). We identified 33.3% (240,269 out of 721,404 peaks) of the 5hmC peaks as being differentially hyperhydroxymethylated in different tissues, which we term as tissue-specific differentially hydroxymethylated regions (tsDhMRs) (Supplementary Fig. 5a, See Methods). Majority of the tsDhMRs are located in intron and distal regions (56.0% and 31.5%, respectively, Supplementary Fig. 5b). We further analyzed tsDhMRs at distal regions and found that they are enriched for H3K4me1 and H3K27ac in corresponding tissues, which are putative enhancer marks (Fig. 4c, d and Supplementary Fig. 5c). Thus, tsDhMRs reside in regions with regulatory potential. In addition, tsDhMRs show higher evolutionary conservation than random regions (Supplementary Fig. 5d), suggesting the importance of such functional elements.

We then investigated how these tsDhMRs may contribute to the gene expression programs of specific tissues. We adopted an existing method to identify linkages between tsDhMRs and putative target genes occurring within a 500-kb window^33^. In total, we identified 2,279 genes whose expression showed significant correlations with 5hmC signals in tsDhMRs (Fig. 4e and Supplementary Fig. 5e). These were genes with tissue-specific expression, such as *CYP11B2* in adrenal glands and *NPPB* in heart. Gene ontology (GO) analysis demonstrated that these genes are enriched in tissue-specific functions, such as learning or memory (brain), kidney epithelium development (kidney), female gonad development (ovary) and sex differentiation (uterus) (Fig. 4f and Supplementary Fig. 5f). Similarly, KEGG pathway analysis revealed that tsDhMR-associated genes participate in tissue-specific functional pathways (Supplementary Fig. 5g). *CYP2C8*, which is involved in the metabolism of xenobiotics, is highly expressed in liver, with liver-specific DhMRs upstream of *CYP2C8* (Fig. 4g). Meanwhile, *PTF1A* plays a vital role in mammalian pancreatic development; and there are pancreas-specific DhMRs around the gene (Supplementary Fig. 5h). In summary, tsDhMRs may contain putative regulatory elements, which specifically affect the expression of nearby functional genes.

### Tissue-specific transcription factors are enriched in tsDhMRs

**Fig. 5:**
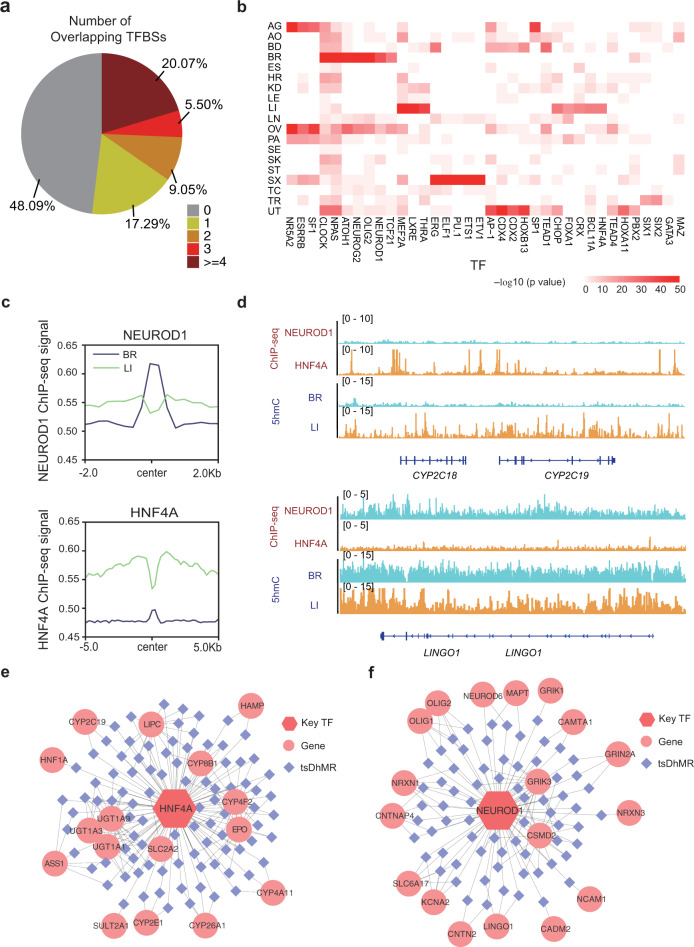
tsDhMRs are enriched for tissue-specific TFBSs. **a** Overlap of distal tsDhMRs with ENCODE TFBSs. **b** The TF motifs enriched in distal tsDhMRs of each tissue. The color scale represents the –log_10_(*P*-value). One-side binomial test (default by homer2). **c** ChIP-seq signals of NEUROD1 and HNF4A around BR-specific DhMRs and LI-specific DhMRs. **d** IGV visualization of 5hmC signals and ChIP-seq signals of NEUROD1 and HNF4A in the brain and liver. **e**, **f** Key TF regulatory networks in the liver (**e**) and brain (**f**). Hexagon represents key TF; rhombi represents tsDhMRs; circles represents regulated genes. Lines linking the hexagon and rhombi indicate that the TF binds to the tsDhMRs, which is supported by TF ChIP-seq data.

We next analyzed potential transcription factor-binding site (TFBS) within tsDhMRs. We found that more than half of the tsDhMRs overlapped with at least one TFBS (Supplementary Fig. 6a), and 20.1% of the distal tsDhMRs even overlapped with no less than four TFBSs (Fig. 5a). We further investigated different transcription factors (TFs) enriched in tsDhMRs derived from each tissue type and found that tissue-specific TFs were significantly enriched in the corresponding tsDhMRs, regardless of H3K4me1/H3K27ac modification (Fig. 5b and Supplementary Fig. 6b, c). Taking NEUROD1 and HNF4A as examples, NEUROD1 plays an important role in the regulation of the differentiation process of various nervous system cells during development, while HNF4A regulates the expression of several hepatic genes. By comparing to ENCODE ChIP-seq data, we found that NEUROD1 specifically binds to brain-specific DhMRs, while HNF4A specifically binds to liver-specific DhMRs (Fig. 5c and Supplementary Fig. 6d). We further used *LINGO1* and *CYP2C18*, which are downstream targets of NEUROD1 and HNF4A in the brain and liver, respectively, to demonstrate the tissue-specific coexistence of 5hmC signals (Fig. 5d). Thus, key tissue-specific TFs may rewire their tissue-specific network through binding to tsDhMRs.

To further understand the regulatory functions, we constructed regulatory networks mediated by HNF4A and NEUROD1 with their corresponding tsDhMRs. We found that HNF4A can regulate genes with tissue-specific expression, such as *CYP4F2*, *CYP8B1*, and *UGT1A9*, via a process potentially mediated by liver-specific DhMRs (Fig. 5e). Within the NEUROD1 network, brain-specific DhMRs regulate *GRIK3, CSMD2* and other genes to perform their brain-specific functions (Fig. 5f). Through network analysis, we illustrated that the key TFs interact with tsDhMRs, which may further affect the expression of tissue-specific functional genes.

### GWAS SNPs are preferentially located within tsDhMRs

**Fig. 6:**
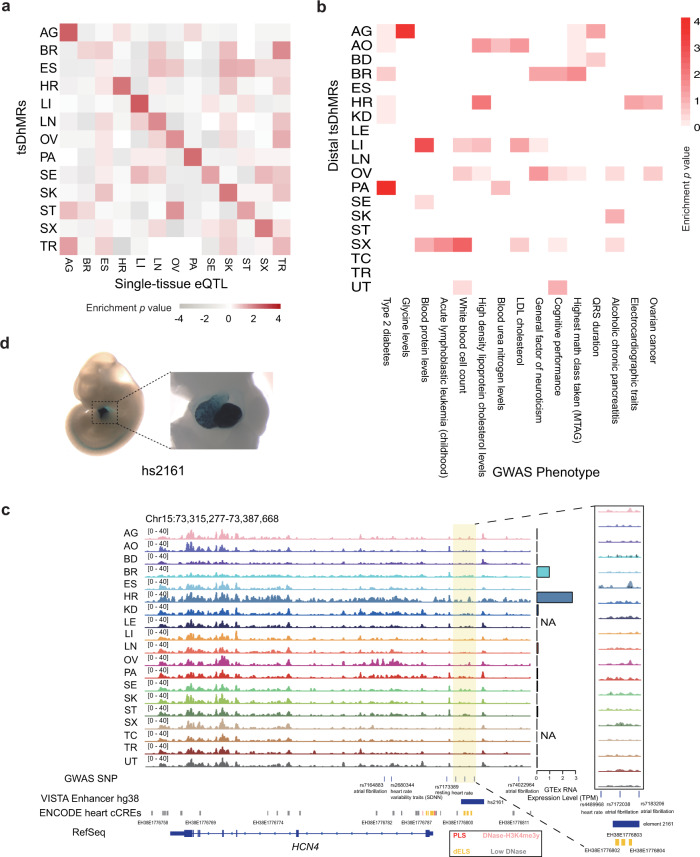
tsDhMRs enrich tissue-specific, phenotypic GWAS SNPs. **a** Overlap of tsDhMRs with single-tissue eQTL SNPs. eQTL SNP data are from GTEx. One-sided (greater) fisher’s exact test. **b** Representative GWAS phenotypes enriched in distal tsDhMRs. One-sided (greater) fisher’s exact test. **c** IGV visualization of the 5hmC signals around *HCN4* on chromosome 15. The locations of GWAS SNPs and VISTA enhancers are also shown. The highlighted region shows the LI-specific DhMRs. **d** In vivo reporter assay of enhancer activity for hs2162. This pictures were obtained from the VISTA enhancer browser^35^.

We next analyzed the potential relationship of tsDhMRs with functional single-nucleotide polymorphisms (SNPs). We found that the tsDhMRs derived from each tissue highly overlapped with the GTEx single-tissue eQTL SNPs (Fig. 6a). In fact, tsDhMRs contain SNPs that are functional in the corresponding tissues. Moreover, tsDhMRs are also enriched for GWAS SNPs^34^ with phenotypes related to the corresponding tissue functions (Fig. 6b and Supplementary Fig. 6e, f), indicating that tsDhMRs reside in regulatory elements that associate with tissue-related diseases. We used several examples to elaborate the findings: (1) SNPs related to electrocardiographic traits and QRS duration were highly enriched in heart-specific DhMRs; (2) SNPs related to metabolite levels and LDL cholesterol were enriched in liver-specific DhMRs; and (3) SNPs related to type 2 diabetes were enriched in pancreas- and adrenal gland-specific DhMRs.

More specifically, we used an example to illustrate the potential mechanism by which distal GWAS SNPs may impact diseases. *HCN4*, which is necessary for the cardiac pacemaking process, is specifically expressed in the heart (Fig. 6c). We found that several heart-related GWAS SNPs were localized in the heart-specific DhMR (Fig. 6c), indicating that the DhMR is associated with heart diseases. Moreover, several enhancers of the heart identified by ENCODE candidate cis-regulatory elements (cCREs) were located in this DhMR (Fig. 6c). We further integrated the VISTA enhancer data^35^, which were validated by transgenic mouse assays, and found an enhancer (hs2161 from VISTA) within the DhMR. This enhancer is specifically expressed in the mouse heart (images from VISTA database) (Fig. 6d). These data confirm that DhMR, located in enhancer, is functional in the heart and is related to heart diseases. These results indicate that dysregulation of tsDhMRs may be involved in human disease pathologies. Collectively, our data show that tsDhMRs could help us understand the function of distal GWAS SNPs in the corresponding tissues.

## Discussion

In this study, we present a base-resolution atlas of 5hmC in human tissues. Hundreds of thousands of 5hmC peaks and millions of 5hmC sites were identified in this dataset, expanding the epigenomic landscape determined by previous large-scale efforts, for example, the ENCODE project. While our work was under review, a 5hmC tissue map of adults of European descent was published, which found that 5hmC is preferentially enriched on tissue-specific gene bodies and enhancers^36^. Thus, tissue-specificity of 5hmC is validated in different human ancestry. Moreover, this study has enabled additional findings: (1) The correlation between 5hmC/5mC levels and gene expression in multiple human tissues has not been directly compared previously. We found that tissue-specifically expressed genes show tissue-specific 5hmC patterns, which is not observed for 5mC; (2) tsDhMRs reside in regions with regulatory potential to control the expression of nearby tissue-specific functional genes; (3) tsDhMRs are enriched with tissue-specific transcription factor-binding sites and rewire the regulatory network of tissue-specific transcription factors; (4) tsDhMRs reside in regulatory elements that associate with tissue-related functional GWAS SNPs.

Our 5hmC tissue map is base-resolution, owning to the use of hmC-CATCH^21^. This feature not only improves the confidence of 5hmC detection (Supplementary Fig. 1e), but also reveals information with regard to the detailed distribution pattern of 5hmC (Fig. 2). For instance, we found a “CAhmCGT” motif that resembles the binding motif of ARNT While approximately half of ARNT binding sites contain 5hmC, only a small part of 5hmC sites contain ARNT binding sites. Thus, ARNT binding only partially explains the 5hmC motif. Among the many possibilities for this observation, other transcription factors could be invovled.

Gene body 5hmC levels are positively correlated with gene expression, especially of genes with tissue-specific expression. Thus, the gene body 5hmC levels may be used to infer gene expression in tissues. This can be particularly useful in some precious clinical samples, where RNA degradation is severe (frozen samples, formalin-fixed paraffin-embedded samples, body fluids and so on). For instance, 5hmC levels in cell-free DNA (cfDNA) have been utilized as biomarkers for cancer diagnosis^21,37–39^. While it is anticipated that healthy and cancerous tissues may have different hydroxymethylomes, cancer-specific 5hmC signatures, which may reflect the gene expression program in different cancers, could be identified for noninvasive cancer diagnosis. Thus, the relative stability of epigenetic modifications makes 5hmC a promising candidate for prediction of gene expression in clinical samples.

Using the hydroxymethylome of multiple tissues, we discovered that approximately one-third of all 5hmC peaks are tissue-specifically hyperhydroxymethylated. We found that tsDhMRs, residing in potential regulatory regions, are positively correlated with gene expression, which is in contrast to the fact that differentially methylated regions (DMRs) show a negative correlation^40^. Moreover, tissue-specific TFs are enriched in tsDhMRs, providing a mechanism by which key TFs may regulate tissue-specific gene expression via tsDhMRs. Based on our identified tsDhMRs, future studies could be designed to illustrate the specific mechanisms by which 5hmC can regulate tissue development and differentiation.

Through integration of GWAS SNP and 5hmC data, we discovered that tsDhMRs were significantly enriched with GWAS SNPs. Our data indicate that tsDhMRs contribute to tissue-related diseases. Although GWAS SNPs are consistent among somatic cells, they may affect tsDhMR 5hmC levels and ultimately lead to dysfunctions of the corresponding tissues. Our analysis provides new insights into the understanding of GWAS data, where distal GWAS SNPs interact with tsDhMRs to regulate target genes. Disruption of tsDhMR 5hmC levels may result in tissue-related disease phenotypes.

Collectively, our data provide a rich resource for understanding the 5hmC landscape in human tissues. The reported human tissue hydroxymethylome adds to the knowledge of how this epigenetic mark may affect tissue-specific differentiation and diseases.

## Methods

### Biospecimen collection

We collected a total of 60 tissue samples from deceased donors who had died of natural and accidental deaths at Zhongshan Hospital (Shanghai, China); we targeted 19 distinct tissues from 6 postmortem Chinese individuals, including 3 males and 3 females. All samples were obtained research consent from the families and informed consent under a protocol approved by the ethics committee of School of Basic Medical Sciences of Fudan University, China. The collected samples were transferred to cryovials for long-term storage at −80°C.

### Preparation of genomic DNA

Genomic DNA was isolated from tissues using the Blood/Cell/Tissue Genomic DNA Extraction Kit (TIANGEN, DP304) following the manufacturer’s specifications. The 5hmC sequencing libraries were constructed with 200 ng of genomic DNA. Briefly, the DNA was fragmented with a ME220 Focused-ultrasonicator (Covaris, ME220) to 300–500 bp. We added a model sequence as a spike-in to the fragmented DNA (Ref, 5hmC and 5fC spike-in, Supplementary Table 3) followed by NEBNext® End Repair Module (NEB) according to the manufacturer’s protocol. Then, the repaired DNA samples were purified by 1.8x AMPure XP (Beckman Coulter) according to the manufacturer’s protocol.

### Hydroxylamine-mediated blocking of endogenous 5fC

Repaired DNA was added to 10 mM O-ethylhydroxylamine (Aldrich, 274992) in 50 μL 100 mM MES buffer (pH 5.0) at 37 °C (850 rpm) for 4 h in a thermomixer (Eppendorf). 5fC-blocked DNA was purified with AMPure XP beads (Beckman Coulter) and Micro Bio-Spin P-6 SSC column (Bio-Rad).

### K_2_RuO_4_-mediated oxidation of genomic DNA and labeling

The 5fC-blocked DNA was denatured in 0.05 M NaOH (total volume 48.5 μL) for 30 min at 37 °C. The reaction was snap cooled on ice-water bath (0 °C) for 5 min. Add 1.5 μL 15 μM K_2_RuO_4_ to the denatured DNA sample and briefly mix the reaction. Leave the oxidation reaction on ice for 1 h. Moreover, using a Bio-Rad Micro Bio-Spin P-6 SSC column (Bio-Rad) to purify the oxidized sample. Then, the purified sample was added to 0.3 mg AI (J&K scientific, 2793949) in 100 μL 20 mM Tris-HCl buffer (pH 8.0) at 37 °C for 18–20 h in a thermomixer (Eppendorf, 850 r.p.m.) and purified by MinElute PCR Purification Kit (QIAGEN).

### Library preparation and click chemistry

The AI-labeled ssDNA was used directly for library preparation with the TELP protocol except PCR amplification^41^. Frist, mixing 28 μL of AI-labeled ssDNA, 1 μL of 10× EX buffer (Takara), 1 μL of 1 mM dCTP (NEB) and 1 μL of terminal deoxynucleotidyl transferase (TDT; NEB) for 37 °C for 35 min, 75 °C for 20 min. Second, the following extension mix to the above-mentioned TDT reaction: 6.2 μL of H_2_O; 0.8 μL of KAPA2G Robust HS (KAPA); 12 μL of 5× KAPA buffer A (KAPA); 4.8 μL of 2.5 mM dNTP (Takara) and 6 μL extension primer (Ex primer, Supplementary Table 3). The extension program was as follows: (i) 95 °C for 3 min; (ii) 47 °C for 1 min, 68 °C for 2 min, 16 cycles and (iii) 72 °C for 10 min. Moreover, using exonuclease I (Exo I) (NEB) digest excess primers at 37 °C for 1 h. Then, the extended DNA was purified by MinElute PCR Purification Kit. Thirdly, mixing 8.4 μL DNA, 0.6 μL adapter containing unique molecular identifier (UMI), 10 μL 2x quick ligation buffer (NEB) and 1 μL Quick T4 ligase (NEB) for 20 °C 1 h. Then the reaction was purified with MinElute PCR Purification Kit. Then, the ligated DNA was added to DBCO-S-S-PEG3-Biotin (Click Chemistry Tools, Cat. No. A112-10) with the final concentration of 400 mM, and incubated in the thermomixer for 1 h at 37 °C (800 rpm), and purified by DNA Clean & Concentrator 5 (Vistech).

### Enrichment of 5hmC-containing DNA

The Dynabeads MyOne Streptavidin C1 (Invitrogen) was used to enrich the biotin-labeled DNA according to the manufacturer’s protocol. Beads were then resuspended and incubated in freshly prepared 50 mM DTT to release the 5hmC-containing strand for 1 h at 37 °C. Then the supernatant was purified with Micro Bio-Spin P-6 Gel Columns (Bio-Rad) to remove DTT. The enriched DNA was amplified with Q5® Hot Start High-Fidelity 2X Master Mix (NEB) according to the manufacturer’s protocol and purified with 1× AMPure XP beads.

### qPCR to detect the enrichment of 5hmC

The qPCR assay had two replicates with SYBR Premix Ex TaqTM II (TAKARA) according to the manufacturer’s instructions. The reaction was performed with Roche LightCycler® 96 Instrument. For spike-in sequences and primers, see Supplementary Table 3.

### Data processing

Illumina sequencing adapters and low-quality reads were removed from raw sequencing data to obtain clean data. We added a sequencing barcode through the hmC-CATCH protocol to mark PCR-duplicated reads. Then, the PCR-duplicated reads were filtered out, and only one read was retained using an in-house script. The final cleaned reads were mapped to hg38 by Bismark (Version: v0.15.0). To enhance the signal-to-noise ratio, we used only read pairs with more than one C-to-T conversion for further analysis.

### Assessing the C-to-T conversion rate of 5hmC sites

We added a model sequence as a spike-in before constructing the hmC-CATCH library, of which one C site was 100% hydroxymethylated. After treatment through the hmC-CATCH protocol, we observed a C-to-T conversion signal in this 5hmC site. We used the *T*/(*C* + *T*) of the sequencing data at this site to estimate the C-to-T conversion rate.

### Identification of 5hmC sites

We used Bismark (Version: v0.15.0) to extract single-base-resolution information. Sites with less than five total bases (NT + NC) or three NTs were discarded for 5hmC calling. Then, we used the binomial distribution with N as the sequencing depth (NC + NT) and p as the normal cytosine conversion rate to assess the probability of observing NT by chance. We considered 5hmC sites with the Holm-Bonferroni method-adjusted *P* < 0.001 and located within a type of tissue 5hmC-enriched region as high-confidence 5hmC sites.

### Identification of 5hmC peaks

Our hmC-CATCH approach could enrich DNA fragments with 5hmC. Here, we used a peak calling method to identify these regions with 5hmC. MACS2 (Version: v2.1.1) was applied to call peaks in each sample with the following command:

“macs2 callpeak -t <5hmC bam> -c <input bam> -g hs -f BAMPE–keep-dup all–outdir <outdir> -n <sample name> ”

### Annotation of 5hmC sites and peaks

The 5hmC sites or peaks were annotated by annotatePeaks.pl (Homer, Version: v4.5), and the “-annStats” parameter was added to quantify the enrichment of genomic elements compared to the background. Then, the log2 ratios of observation to expectation in all tissues were plotted as a bar chart by ggplot2 (R package, Version v3.5.1).

### tSNE cluster of the global 5hmC signals

The whole hg38 genome was first cut into 10-kb non-overlapping bins. Then, the read counts in each 10-kb bin of all samples were calculated by “Bedtools multicov” (bedtools, Version: v2.27.1). After normalizing the sequencing depth (DESeq2, R package) and batch effect (limma, R package, Version v3.5.1), we performed tSNE clustering (Rtsne, R package, Version v3.5.1) to reduce the high-dimensional data to two dimensions. ggplot2 (R package, Version v3.5.1) was used to visualize the data.

### Analysis of the correlation of gene body 5hmC and 5mC

The RPKM values of all protein-coding genes were calculated and normalized as mentioned above. Spearman’s correlation coefficients between the RPKM values and 5hmC/5mC signals in matched tissues were calculated by R (cor, method = “spearman”, Version v3.5.1).

### Identification of genes with tissue-specific expression

We used GTEx gene expression data (RNASeQCv1.1.9_gene_median_tpm) to identify the genes with tissue-specific expression that were defined as being highly expressed in one tissue. We first filtered out the genes with a mean TPM <1, which was regarded as low expression in all tissues. Then, we calculated the fold changes in the expression of protein-coding genes in one tissue over the mean values for other tissues. We ordered the genes according to fold change levels, and the top 300 in each tissue were regarded as genes with tissue-specific expression. To ensure that the top 300 genes with tissue-specific expression in each tissue were significantly more highly expressed than others, we filtered out the genes with fold changes lower than 2.

### Identification of tissue-specific differential 5hmC regions

We first merged all 5hmC peaks from the 60 samples to obtain the total peaks using “Bedtools merge” (bedtools, Version: v2.27.1). Then, the read counts in each merged peak of all samples were calculated by “Bedtools multicov” (bedtools, Version: v2.27.1) and normalized as mentioned above. We merged all biological replicates from the same tissue to enhance the signals. We used the Poisson distribution to estimate the *P*-value of each peak in a tissue. The probability of read counts in each peak of one tissue was estimated by the one-tail Poisson distribution with the parameter *λ* as the mean for other tissues. The *P*-values were further adjusted by the Bonferroni method. The peaks with adjusted *P*-values < 0.05 and fold changes >2 were regarded as significant tissue-specific differential 5hmC regions.

### Identification of tsDhMR-associated genes

We adapted a method to link tsDhMRs to putative genes^33^. We first identified all possible genes linked to tsDhMRs by searching any TSS of GENCODE (Version: v28) protein-coding genes within 500 kb of the tsDhMRs. Then, we calculated the Pearson correlation coefficients between normalized 5hmC signals and gene expression (TPM). To avoid spurious associations, we used cor.test (R, Version v3.5.1) to assess the stochastic links. Finally, we required that the confident links had a Pearson correlation over 0.8 and *P*-value lower than 0.05.

### Motif-enrichment analysis

For each tissue, we used findMotifsGenome.pl (Homer, Version: v4.5) to find the motifs enriched in tsDhMRs with the following command:

“findMotifsGenome.pl <tsDhMRs bed> hg38 <output> -p 5”

### Analysis of tsDhMRs associated with GWAS SNPs

We downloaded previously published GWAS datasets from the GWAS catalog^34^. Then, we calculated the associations of GWAS phenotypes and tsDhMRs. A GWAS phenotype always be associated with multiple SNPs. For each phenotype, we used Fisher’s test to compute the significance and odds ratios between the phenotype-associated SNPs and tsDhMRs.

### Reporting summary

Further information on research design is available in the Nature Research Reporting Summary linked to this article.

## Supplementary information

Supplementary information

Reporting Summary

## Data Availability

The data that support this study are available from the corresponding authors upon reasonable request. Sequencing data have been deposited into the Gene Expression Omnibus (GEO) under the accession number GSE134078. This study used previously published data including BS-seq (GSE119981, GSE112356, GSE59395)^42–44^, ChIP-seq data for histone modifications (GSE59395, GSE16256)^44,45^ and DNase-seq (GSE18927)^45^, RNA-seq data from GTEx (https://gtexportal.org/home/datasets)^30^, and the GWAS datasets from the NHGRI-EBI GWAS catalog (https://www.ebi.ac.uk/gwas/docs/file-downloads)^34^. All data related to the manuscript have been published.

## Code availability

The analysis scripts have been deposited into GitHub [https://github.com/herb-BO/hmC_CATCH].
