# Supplementary material for: Tissue-specific 5-hydroxymethylcytosine landscape of the human genome
Source: Nat Commun. 2021 Jul 12;12:4249. doi: 10.1038/s41467-021-24425-w (PMC8275684; doi:10.1038/s41467-021-24425-w)
Supplement: Supplementary file 1 — Supplementary information [file 41467_2021_24425_MOESM1_ESM.pdf]

Supplementary information

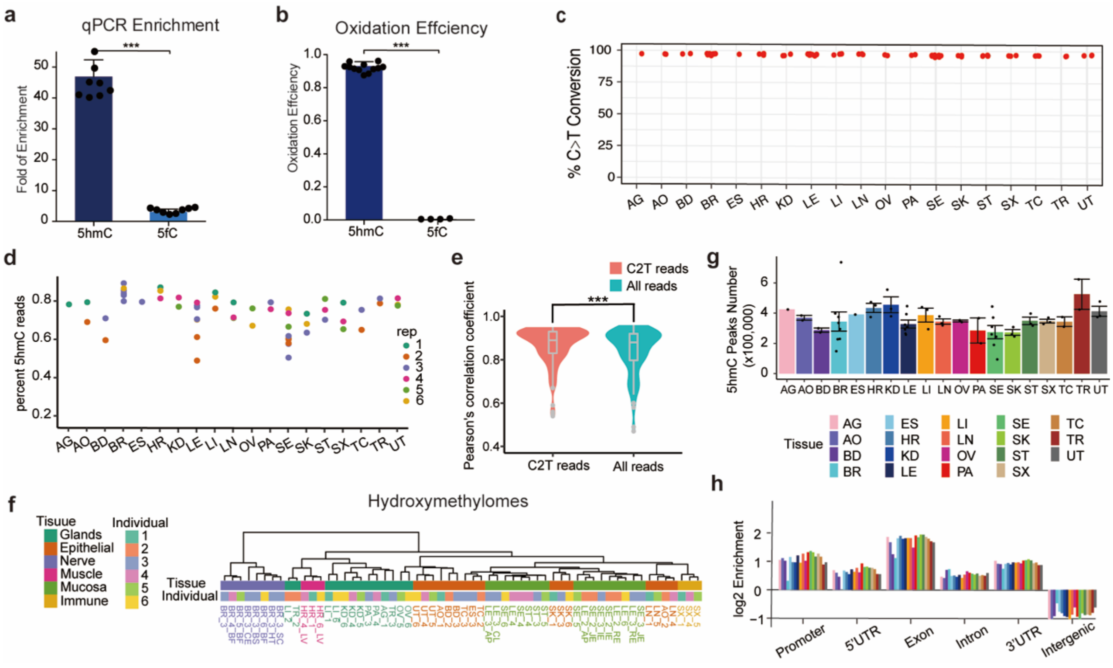

Extended Data Figure 1. hmC-CATCH data from different tissues are high quality and reproducible. a. Enrichment of spike-in probes detected by qPCR (*** represents *P* value < 0.001, *P* value: $3.46x10^{-8}$, one-side paired t-test, n=8 biologically independent samples, data are represented as mean values +/- SD). **b.** The oxidation efficiency in 5hmC and 5fC spike-in probes by high-throughput sequencing (*** represents *P* value < 0.001, *P* value: $1.53.46x10^{-19}$, one-side t-test, n_{5hmC}=12 biologically independent samples, n_{5fC}=4 biologically independent samples, data are represented as mean values +/- SD). **c.** C-to-T conversion rates of spike-in probes in all samples. **d.** Cytosine-to-thymine conversion rates in all samples. **e.** Pearson's correlation of biological replicates using total reads or filtered reads (*** represents *P* value< 0.001, *P* value: $1.06x10^{-7}$, one-side paired t-test , n=60 biologically independent samples, Data are represented as mean values; box limits indicate the first and third quantiles). **f.** Hierarchical clustering

of all samples using the first 20 principal components. **g.** Numbers of 5hmC peaks in all tissues (Data are represented as mean values +/- SD). The number of samples are shown in Supplementary Table 2. **h.** Enrichment of 5hmC peaks in different genomic features in all tissues.

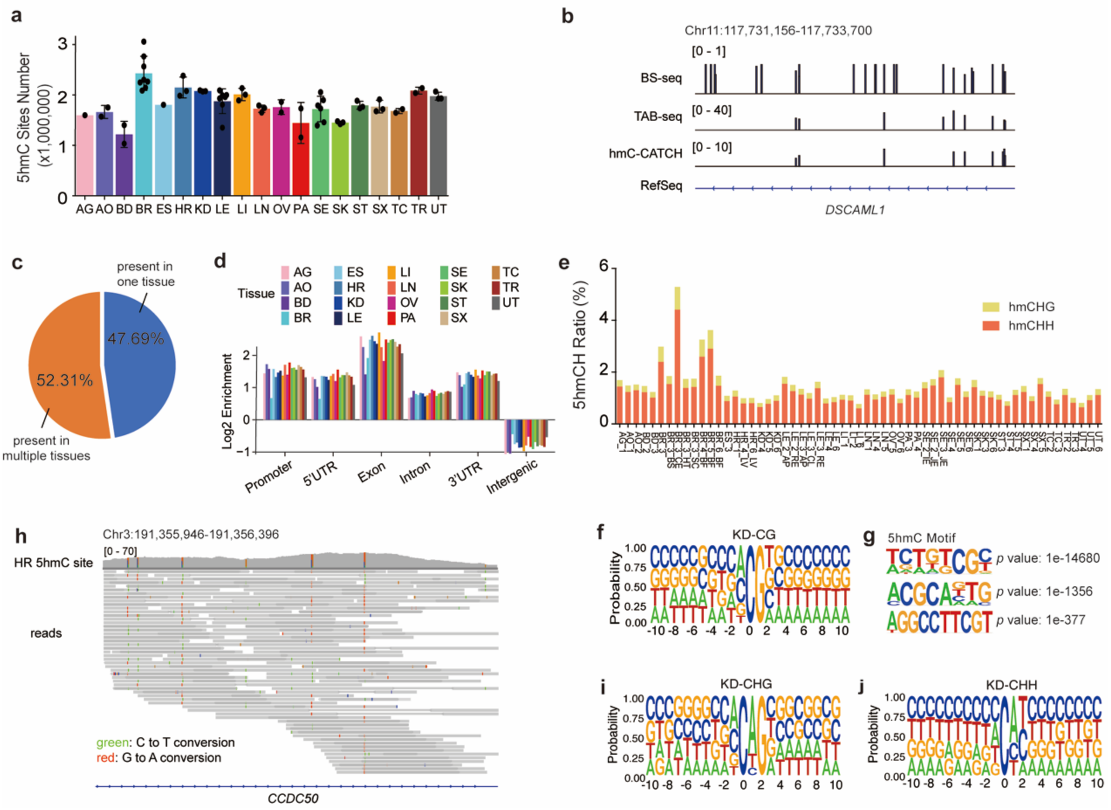

Extended Data Figure 2. Brief introduction of 5hmC sites. a. Numbers of 5hmC sites in all tissues. Error bar represents standard deviation (Data are represented as mean values +/- SD). The number of samples are shown in Supplementary Table 2. **b.** An examples of the single-base resolution 5hmC/5mC data on chromosome 11 in brain tissue. **c.** Proportions of 5hmC sites present in unique tissue or multiple tissues. **d.** Enrichment of 5hmC sites in different genomic features of all tissues. **e.** The proportion of 5hmCHH and 5hmCHG. **f.** Sequence context ± 10 bp around 5hmCG sites in kidney. **g.** The 5hmC motif identified by HOMER. **h.** IGV visualization of 5hmC signals at each read on chromosome 3 of heart. Red dot represents G-to-A conversion and the green dot represent C-to-T conversion. **i-j.** Sequence context ± 10 bp around 5hmCHG (i) and 5hmCHH (j) sites in kidney.

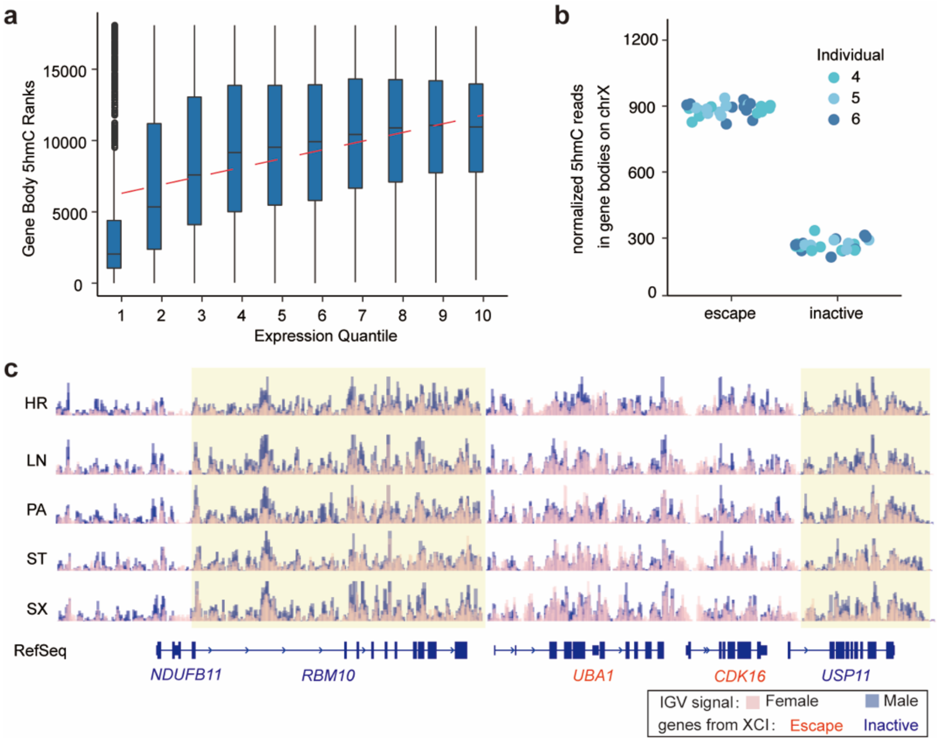

Extended Data Figure 3. Chromosome X inactivation is associated with gene body 5hmC level. a. Correlation of mean ranks of gene body 5hmC levels and gene expression levels in heart tissue. Genes were divided into ten groups according to their expression levels (Data are represented as mean values; box limits indicate the first and third quantiles, n = 2,020 genes). **b.** The normalized read counts in escaped and inactivated genes. **c.** Overlapped IGV visualization of the normalized 5hmC signals of male and female tissues on chromosome X. The 5hmC signals of male tissues are multiplied by 2. Male samples are colored blue, and female samples are colored pink. The highlighted genes are inactive genes in XCI, and *UBA1* and *CDK16* escaped from XCI.

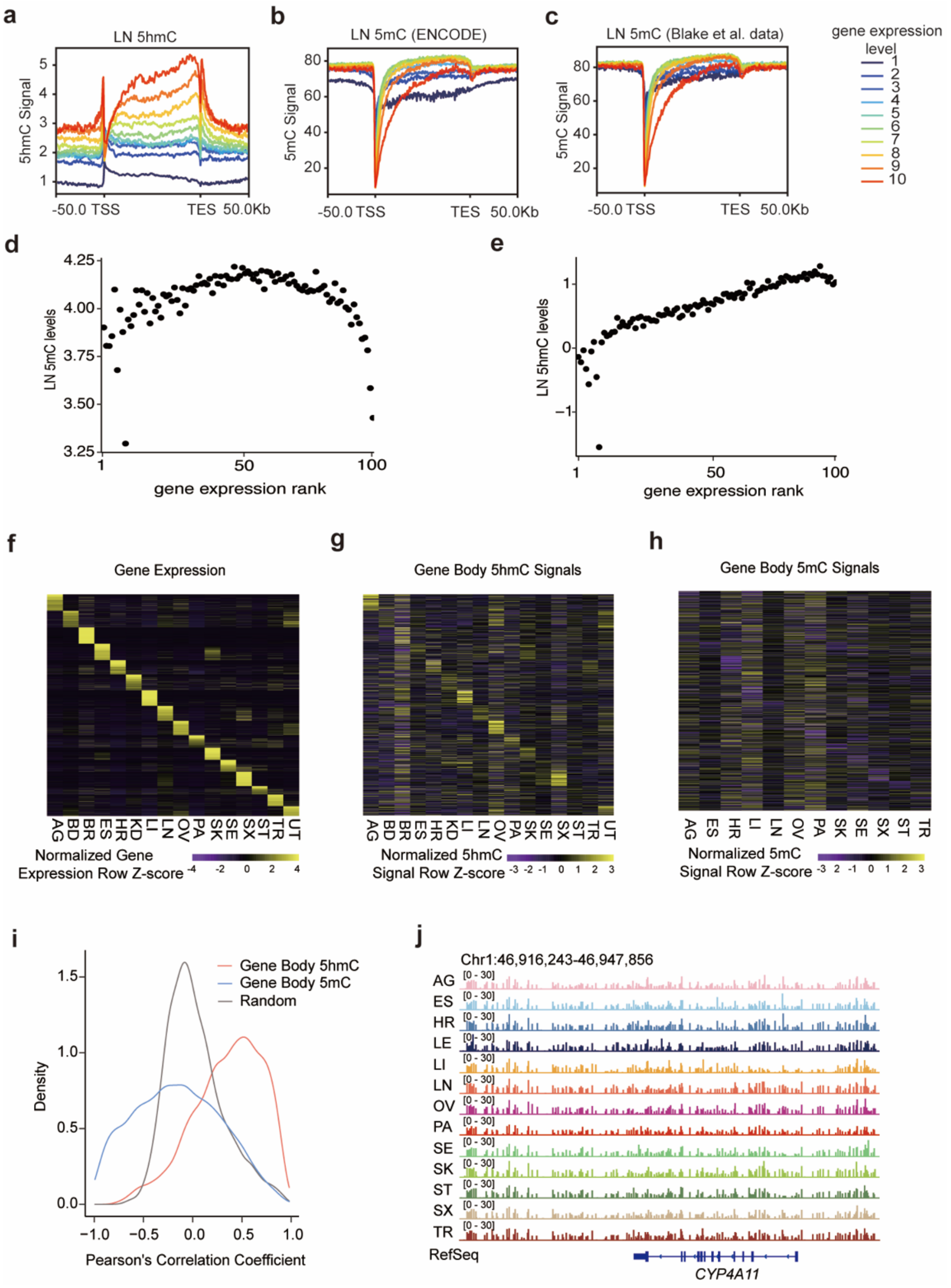

Extended Data Figure 4. Gene body 5hmC is positively correlated with gene expression.

a-c. The correlation between 5hmC level (a), 5mC level downloaded from ENCODE (b) or 5mC level downloaded from Blake et al. and gene expression level which is classified into ten levels according to expression level in lung. **d,e.** The correlation of 5mC(d) and 5hmC(e) level with gene expression in lung, in which genes are classified

into 100 groups according to their expression. **f.** Heatmap displaying the expression levels of tissue-specifically expressed genes. Gene expression data of matched tissue samples were downloaded from the GTEx project. **g, h.** Heatmap displaying the normalized gene body 5hmC (g) and 5mC (h) signals of tissue-specifically expressed genes. The order of the row is identical to that in (f). The 5mC data were downloaded from ENCODE. **i.** Distribution of Pearson's correlation between gene body 5hmC (5mC) levels and tissue-specific gene expression levels across all tissues. These tissue-specific genes are identical to that in (f). **j.** IGV visualization of the 5mC signals at the gene body of *CYP4A11* and nearby regions.

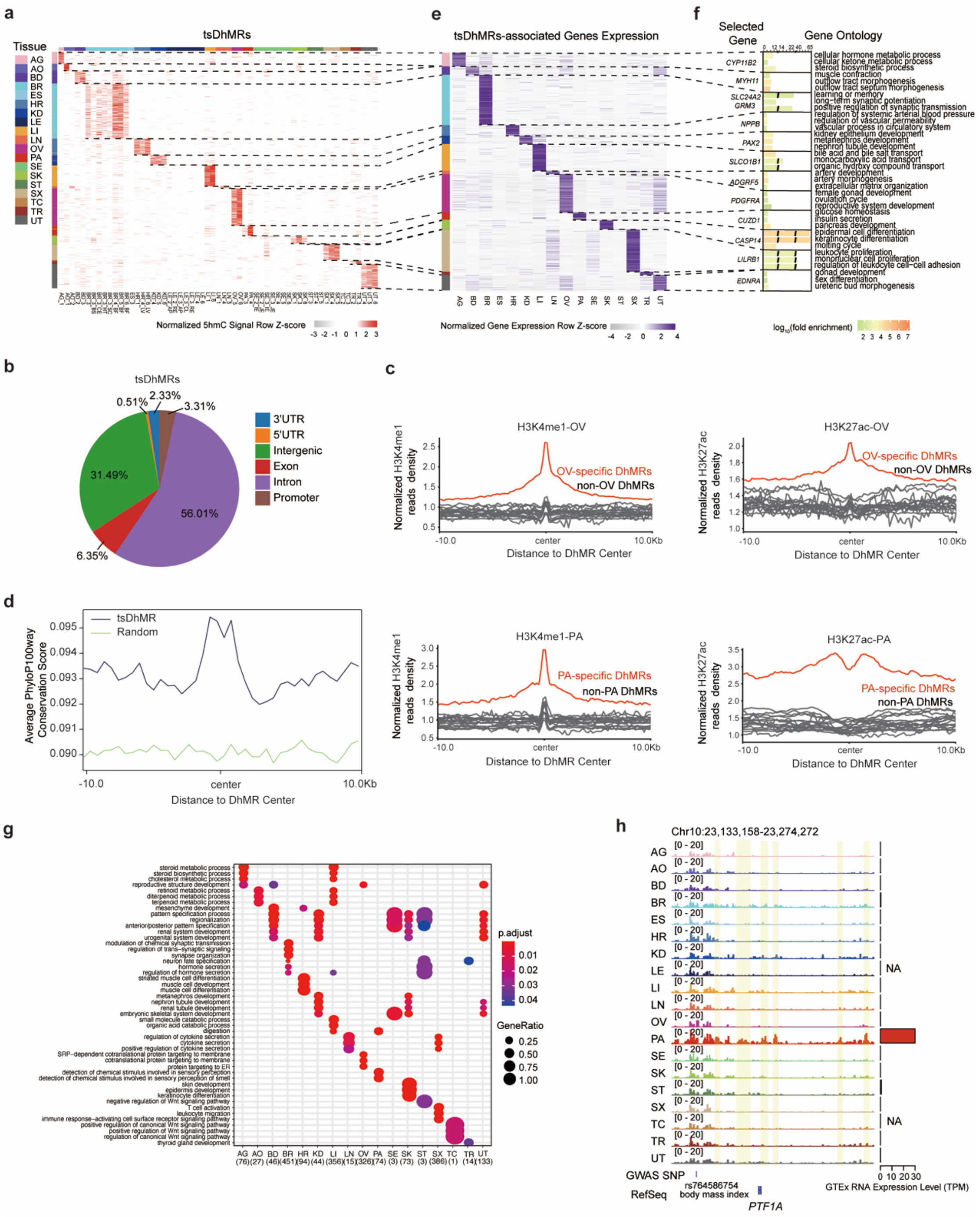

Extended Data Figure 5. Function of tsDhMRs. a. Heatmap showing the normalized 5hmC signals in all tsDhMRs. **b.** Pie chart showing the percentage of tsDhMRs in each genomic element. **c.** Profiles of H3K27ac and H3K4me1 modifications around distal tsDhMRs in the pancreas (PA) and ovary (OV). Red represents the tissue type mentioned in the title of each panel and grey denotes the rest of the tissue types. **d.**

Average phyloP100way conservation scores around tsDhMRs. Higher scores indicate greater conservation. **e.** Heatmap showing the expression of genes associated with tsDhMRs within ± 500 kb. **f.** GO enrichment and representative genes of tsDhMR-associated genes. **g.** KEGG pathway enrichment of tsDhMR-associated genes. **h.** IGV visualization of the 5hmC signals nearby *PTF1A* on chromosome 10. The highlighted regions are pancreas-specific DhMRs.

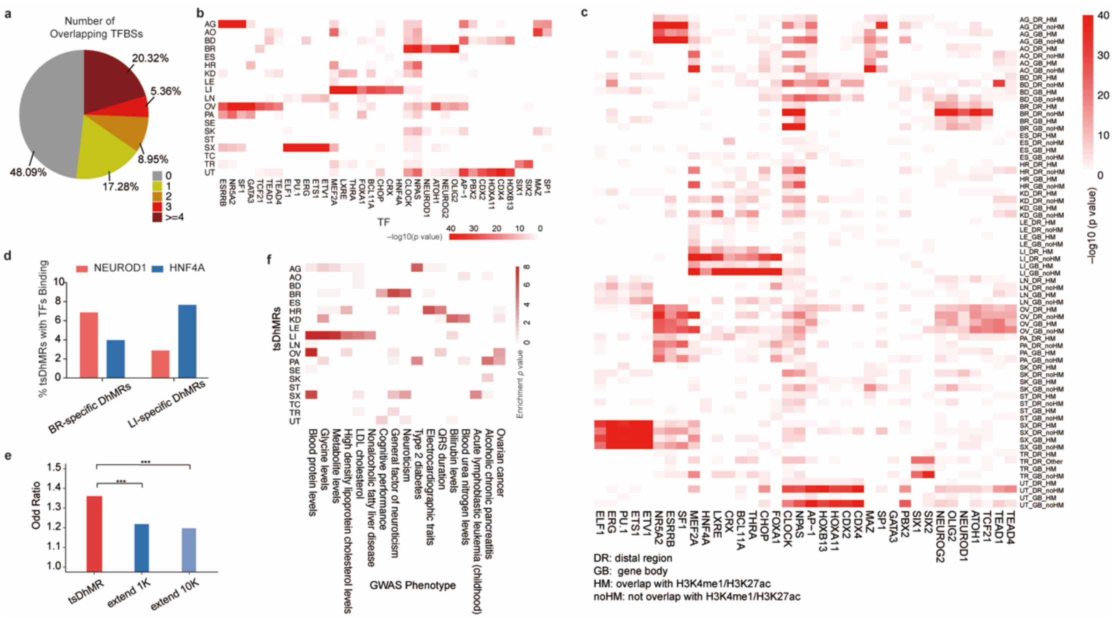

Extended Data Figure 6. tsDhMRs enriched with TFs and GWAS SNPs. a. Overlap of tsDhMRs with ENCODE TFBSs. **b.** The TF motifs enriched in tsDhMRs of each tissue. The color scale represents the $-\log_{10}$ (P value). One side binomial test (default by homer2). **c.** The TF motifs enriched in four categories tsDhMRs: (1) with H3K4me1/H3K27ac and within the gene body (GB_HM); (2) with H3K4me1/H3K27ac but outside the gene body (DR_HM); (3) without H3K4me1/H3K27ac but within the gene body(GB_noHM); and (4) without H3K4me1/H3K27ac and outside the gene body (DR_noHM). One side binomial test (default by homer2). **d.** Proportions of NERUOD1 and HNF4A ChIP-seq peaks in BR-tsDhMR or LI-tsDhMR. **e.** Overlap of GWAS SNPs in tsDhMRs or nearby regions. Chi-squared Test, *** represents P values < 0.0001, n=240,269 regions, tsDhMRs vs extend 1k P value: 5.06×10^{-7}; tsDhMRs vs extend 10k P value: 1.57×10^{-10}. **f.** Motifs enriched in tsDhMRs of each tissue. The color scale represents the $-\log_{10}$ (P value). One-side (greater) fisher's exact test.

Supplementary Table 1 Sample information

Individual	Gender	Age	Race	Nationality	Number of tissue sample	Cause of death	Tissue sample
1	male	76	Asian	Han	7	natural death	AG_1, AO_1, HR_1, LI_1, LN_1, SK_1, SX_1
2	male	24	Asian	Han	9	accidental deaths	AO_2, BD_2, LE_2_AP, LE_2_RE, LI_2, SE_2_IE, SE_2_JE, TC_2, TR_2
3	male	25	Asian	Han	17	accidental deaths	BD_3, BR_3, BR_3_BS, BR_3_CE, BR_3_HT, BR_3_SC, ES_3, LE_3_CL, LE_3_AP, LE_3_RE, PA_3, SE_3_IE, SE_3_JE, SK_3, ST_3, TC_3, TR_3
4	female	44	Asian	Han	10	accidental deaths	BR_4_BF, HR_4_LV, KD_4, LE_4, LN_4, PA_4, SE_4, ST_4, SX_4, UT_4
5	female	30	Asian	Han	8	accidental deaths	BR_5_BF, KD_5, LN_5, OV_5, SE_5, ST_5, SX_5, UT_5
6	female	22	Asian	Han	9	accidental deaths	BR_6_BF, HR_6_LV, KD_6, LE_6, LI_6, OV_6, SE_6, SK_6, UT_6

Supplementary Table 2 Tissues information

Tissue name	Abbreviation	Number
Adrenal gland	AG	1
Aorta	AO	2
Bladder	BD	2
Brain	BR	8
Esophagus	ES	1
Heart	HR	3
Kidney	KD	3
Large Intestine	LE	7
Liver	LI	3
Lung	LN	3
Ovary	OV	2
Pancreas	PA	2
Small Intestine	SE	7
Skin	SK	3
Stomach	ST	3
Spleen	SX	3
Trachea	TC	2
Thyroid	TR	2
Uterus	UT	3

Supplementary Table 3 High-throughput sequencing information

name	rawdata	mappable reads	mapping ratio	unique reads	unique ratio	type
AG_1	185,991,191	83,146,981	52.7	45,549,390	54.78%	glands
AO_1	174,189,243	87,982,443	60.1	42,787,077	48.63%	epithelial
AO_2	137,841,089	78,199,279	64.8	49,453,052	63.24%	epithelial
BD_2	147,618,906	86,402,991	66.7	52,280,865	60.51%	epithelial
BD_3	118,970,470	72,108,880	68.5	44,911,292	62.28%	epithelial
BR_3	136,529,099	86,750,430	72.3	69,600,685	80.23%	nerve
BR_3_BS	114,687,615	68,132,608	68.4	51,981,961	76.30%	nerve
BR_3_CE	134,177,900	88,013,168	75.2	74,504,470	84.65%	nerve
BR_3_HT	117,459,343	70,017,303	68.1	55,747,211	79.62%	nerve
BR_3_SC	120,502,699	75,012,036	71	57,785,219	77.03%	nerve
BR_4_BF	172,035,355	106,612,995	78.2	91,590,006	85.91%	nerve
BR_5_BF	150,835,491	88,997,313	74	78,302,867	87.98%	nerve
BR_6_BF	151,704,902	92,617,478	71.1	65,919,416	71.17%	nerve
ES_3	110,861,079	72,625,525	76.5	58,168,846	80.09%	epithelial
HR_1	98,440,175	61,411,294	68.5	51,090,962	83.19%	muscle
HR_4_LV	85,414,580	57,653,402	76.7	48,259,749	83.71%	muscle
HR_6_LV	99,476,821	62,085,293	73.2	45,280,937	72.93%	muscle
KD_4	112,828,678	75,849,977	75.8	60,720,440	80.05%	glands
KD_5	112,705,901	71,964,723	74.9	62,941,770	87.46%	glands
KD_6	160,340,350	85,933,724	64	38,727,870	45.07%	glands
LE_2_AP	116,415,625	70,315,704	68.2	62,690,098	89.16%	mucosa
LE_2_RE	119,678,917	66,512,511	66.4	52,101,531	78.33%	mucosa
LE_3_AP	111,021,883	66,983,639	68.8	48,412,865	72.28%	mucosa
LE_3_CL	120,017,355	77,612,089	74.8	62,263,556	80.22%	mucosa
LE_3_RE	139,757,558	86,425,081	70.3	50,561,528	58.50%	mucosa
LE_4	137,971,674	81,592,960	67.6	48,727,604	59.72%	mucosa
LE_6	122,469,633	76,044,389	71.9	45,914,372	60.38%	mucosa
LI_1	103,690,563	64,755,555	70.1	52,516,434	81.10%	glands
LI_2	83,673,574	53,535,275	74.3	46,069,291	86.05%	glands
LI_6	95,282,148	58,912,563	71.7	49,400,292	83.85%	glands
LN_1	105,102,831	67,041,882	70.5	50,470,109	75.28%	epithelial
LN_4	157,805,473	92,356,369	66.6	44,630,622	48.32%	epithelial
LN_5	129,987,987	79,311,439	68.5	60,192,879	75.89%	epithelial
OV_5	118,506,060	70,290,084	67.8	51,031,746	72.60%	glands
OV_6	97,502,853	65,352,129	78.9	56,782,463	86.89%	glands
PA_3	124,058,649	79,259,219	71.8	55,046,397	69.45%	glands
PA_4	98,386,719	55,096,999	63.9	30,316,702	55.02%	glands
SE_2_IE	109,395,453	67,257,961	71.9	55,884,369	83.09%	mucosa
SE_2_JE	194,228,176	103,581,017	60.8	75,412,721	72.81%	mucosa
SE_3_IE	102,643,168	55,404,758	62.2	31,254,876	56.41%	mucosa
SE_3_JE	126,502,196	77,524,341	70.5	48,553,208	62.63%	mucosa
SE_4	123,520,769	77,422,024	71.1	52,616,442	67.96%	mucosa
SE_5	160,889,179	82,962,528	61.1	46,532,569	56.09%	mucosa

SE_6	131,439,727	68,939,254	61.2	41,344,263	59.97%	mucosa
SK_1	183,306,648	84,758,833	54.1	41,334,675	48.77%	epithelial
SK_3	132,128,743	77,691,080	68.6	47,678,919	61.37%	epithelial
SK_6	132,922,079	73,533,033	65.1	42,002,923	57.12%	epithelial
ST_3	115,297,511	77,513,575	75.8	56,848,885	73.34%	mucosa
ST_4	93,259,240	58,165,958	69.9	43,745,772	75.21%	mucosa
ST_5	116,143,703	61,699,993	61.1	47,869,404	77.58%	mucosa
SX_1	102,990,683	64,119,879	69.9	50,064,255	78.08%	immune
SX_4	137,632,167	83,274,034	69.4	50,978,378	61.22%	immune
SX_5	149,736,111	78,982,847	60.1	50,962,523	64.52%	immune
TC_1	145,275,582	73,894,451	58.9	51,437,174	69.61%	epithelial
TC_2	109,143,404	60,851,581	66	50,498,476	82.99%	epithelial
TC_3	112,902,388	77,844,624	79.2	58,180,168	74.74%	epithelial
TR_2	102,849,553	63,884,161	71.2	56,098,675	87.81%	glands
TR_3	126,807,528	89,157,247	81.1	74,060,720	83.07%	glands
UT_4	108,944,261	72,479,546	75.5	58,312,003	80.45%	epithelial
UT_5	110,762,954	68,940,888	69.7	54,297,613	78.76%	epithelial
UT_6	136,732,278	76,559,981	63.1	43,206,001	56.43%	epithelial

Supplementary Table 4 Model sequences and primers

ID NO.	Sequence (5'-3')	Annotation
1	CTACGCAAACTGGCTGTCAAAGTAA CTGACCAGATCTCTCGGCTCTCTTG AGGCTACTGAGTTATCATGGACGCT A CCTCACAG	Ref spike-in, dsDNA
2	CTACGCAAACTGGCTGTCAA	qPCR-Ref-F
3	CTGTGAGGTAGCGTCCATGA	qPCR-Ref-R
4	CATGAGTGCCCTCAGCAGTAAGTAA CTGACCAGATCTCTCGTGCCTCTTG AGGCTACTGAGTTATCCAACCTTTA GGAGCCATGCATCGATAGCATCCG5 fCCACAGGCAGTGAGGCTACTGAGT CATGCACGCAGAAAGAAATAGC	5fC spike-in,dsDNA
5	CATGAGTGCCCTCAGCAGTA	qPCR-5fC-F
6	CATGGCTCCTAAAGGTTGGA	qPCR-5fC-R
7	TATAACCCGACGACTCGACCAGTAA CTGACCAGATCTCTCGTGCCTCTTG AGGCTACTGAGTTAAGTGCAACATT GGGGCTAACCATCGATAGCATCCG5 hmCCACAGGCAGTGAGGCTACTGA GTCAGGCCATTGATGCATCTTTCCG AC	5hmC spike-in,dsDNA
8	TATAACCCGACGACTCGACC	qPCR-5hmC-F
9	GTTAGCCCCAATGTTGCACT	qPCR-5hmC-R
10	GTTCAGACGTGTGCTCTTCCGATCT GGGGGGGGG	Ex primer
