# Supplementary material for: Tissue-specific 5-hydroxymethylcytosine landscape of the human genome
Source: Nat Commun. 2021 Jul 12;12:4249. doi: 10.1038/s41467-021-24425-w (PMC8275684; doi:10.1038/s41467-021-24425-w)
Supplement: Supplementary file 2 — Reporting Summary [file 41467_2021_24425_MOESM2_ESM.pdf]

nature research

Corresponding author(s): Chengqi Yi

Last updated by author(s): Mar 1, 2021

Reporting Summary

Nature Research wishes to improve the reproducibility of the work that we publish. This form provides structure for consistency and transparency in reporting. For further information on Nature Research policies, see our Editorial Policies and the Editorial Policy Checklist.

Statistics

For all statistical analyses, confirm that the following items are present in the figure legend, table legend, main text, or Methods section.

n/a	Confirmed	
☐	☒	The exact sample size (n) for each experimental group/condition, given as a discrete number and unit of measurement
☐	☒	A statement on whether measurements were taken from distinct samples or whether the same sample was measured repeatedly
☐	☒	The statistical test(s) used AND whether they are one- or two-sided<br>*Only common tests should be described solely by name; describe more complex techniques in the Methods section.*
☐	☒	A description of all covariates tested
☐	☒	A description of any assumptions or corrections, such as tests of normality and adjustment for multiple comparisons
☐	☒	A full description of the statistical parameters including central tendency (e.g. means) or other basic estimates (e.g. regression coefficient) AND variation (e.g. standard deviation) or associated estimates of uncertainty (e.g. confidence intervals)
☐	☒	For null hypothesis testing, the test statistic (e.g. F, t, r) with confidence intervals, effect sizes, degrees of freedom and P value noted<br>*Give P values as exact values whenever suitable.*
☒	☐	For Bayesian analysis, information on the choice of priors and Markov chain Monte Carlo settings
☒	☐	For hierarchical and complex designs, identification of the appropriate level for tests and full reporting of outcomes
☐	☒	Estimates of effect sizes (e.g. Cohen's d, Pearson's r), indicating how they were calculated

Our web collection on statistics for biologists contains articles on many of the points above.

Software and code

Policy information about availability of computer code

Data collection	All sequencing data were collected by illumina HiSeq X Ten.
Data analysis	The cleaned reads were mapped to hg38 by Bismark (Version: v0.15.0). Using MACS2(V2.1.1.20160309) to identify 5hmC enriched regions. The 5hmC sites or peaks were annotated by annotatePeaks.pl (Homer, Version: v4.5).We first merged all 5hmC peaks from the 60 samples to obtain the total peaks using "Bedtools merge" (bedtools, Version: v2.27.1). Then, the read counts in each merged peak of all samples were calculated by "Bedtools multicov" (bedtools, Version: v2.27.1). After normalizing the sequencing depth (DESeq2, R package V.3.5.1) and batch effect (limma, R package V.3.5.1), we performed tSNE clustering (Rtsne, R package V.3.5.1) to reduce the high-dimensional data to two dimensions. ggplot2 (R package V.3.5.1) was used to visualize the data. The analysis scripts have been deposited into GitHub (https://github.com/herb-BO/hmC_CATCH).

For manuscripts utilizing custom algorithms or software that are central to the research but not yet described in published literature, software must be made available to editors and reviewers. We strongly encourage code deposition in a community repository (e.g. GitHub). See the Nature Research guidelines for submitting code & software for further information.

Data

Policy information about availability of data

All manuscripts must include a data availability statement. This statement should provide the following information, where applicable:

- Accession codes, unique identifiers, or web links for publicly available datasets
- A list of figures that have associated raw data
- A description of any restrictions on data availability

Sequencing data have been deposited into the Gene Expression Omnibus (GEO) under the accession number GSE134078. (https://www.ncbi.nlm.nih.gov/geo/query/acc.cgi?acc=GSE134078). The human brain 5mC data were downloaded from the study (GEO number: GSE119981,https://www.ncbi.nlm.nih.gov/geo/query/

acc.cgi?acc=GSE119981), and this data was used to display single-base 5mC sites. The BS-seq data from Blake et al. were downloaded from the study (GEO number: GSE112356,https://www.ncbi.nlm.nih.gov/geo/query/acc.cgi?acc=GSE112356) and the rest of 5mC data of human tissues were downloaded from the ENCODE project (GEO number: GSE59395,https://www.ncbi.nlm.nih.gov/geo/query/acc.cgi?acc=GSE59395), and both of these 5mC data were used to explore the correlation between 5mC signals and gene expression level. Previously published ChIP-seq data for histone modifications and DNase-seq were acquired from the ENCODE Project (GEO number: GSE59395,https://www.ncbi.nlm.nih.gov/geo/query/acc.cgi?acc=GSE59395, GSE16256,https://www.ncbi.nlm.nih.gov/geo/query/acc.cgi?acc=GSE16256, and GSE18927,https://www.ncbi.nlm.nih.gov/geo/query/acc.cgi?acc=GSE18927), and these data were used to explore the potential regulatory function of tsDhMRs. RNA-seq data from GTEx (https://gtexportal.org/home/datasets), and the GWAS datasets from the NHGRI-EBI GWAS catalog (https://www.ebi.ac.uk/gwas/docs/file-downloads). All data related to the manuscript have been published.

Field-specific reporting

Please select the one below that is the best fit for your research. If you are not sure, read the appropriate sections before making your selection.

☒ Life sciences ☐ Behavioural & social sciences ☐ Ecological, evolutionary & environmental sciences

For a reference copy of the document with all sections, see nature.com/documents/nr-reporting-summary-flat.pdf

Life sciences study design

All studies must disclose on these points even when the disclosure is negative.

Sample size	We can only obtain 60 tissue samples, and all of them are used for this research.
Data exclusions	No data exclusions.
Replication	We do not have technical replication, but we have biological replication on different people in the same organization, to verify the reliability of the results.
Randomization	We group the experiments according to the sample acquisition batch. This technique is very stable, and the batch effects of this study are negligible through bioinformatics analysis. Thus, it is not relevant to our study.
Blinding	The investigators were blinded to group allocation during data collection and data analysis.

Reporting for specific materials, systems and methods

We require information from authors about some types of materials, experimental systems and methods used in many studies. Here, indicate whether each material, system or method listed is relevant to your study. If you are not sure if a list item applies to your research, read the appropriate section before selecting a response.

Materials & experimental systems

n/a	Involved in the study	
☒	☐	Antibodies
☒	☐	Eukaryotic cell lines
☒	☐	Palaeontology and archaeology
☒	☐	Animals and other organisms
☐	☒	Human research participants
☒	☐	Clinical data
☒	☐	Dual use research of concern

Methods

n/a	Involved in the study	
☒	☐	ChIP-seq
☒	☐	Flow cytometry
☒	☐	MRI-based neuroimaging

Human research participants

Policy information about studies involving human research participants

Population characteristics	We collected samples from 6 Chinese donors of Han people, including 3 males(Individual 1:age 76, Individual 2:age 24, Individual 3:age 25) and 3 females(Individual 4:age 44, Individual 5:age 30, Individual 6:age 22).
Recruitment	We collected the tissue samples from deceased donors who had died of natural and accidental deaths at Zhongshan Hospital (Shanghai, China).Only normal tissue is selected, because disease can cause changes the landscape of the hydroxymethylome .
Ethics oversight	The ethics committee of School of Basic Medical Sciences of Fudan University, China

Note that full information on the approval of the study protocol must also be provided in the manuscript.
